# Myricetin Alleviates Pathological Cardiac Hypertrophy via TRAF6/TAK1/MAPK and Nrf2 Signaling Pathway

**DOI:** 10.1155/2019/6304058

**Published:** 2019-12-06

**Authors:** Hai-han Liao, Nan Zhang, Yan-yan Meng, Hong Feng, Jing-jing Yang, Wen-jin Li, Si Chen, Hai-ming Wu, Wei Deng, Qi-zhu Tang

**Affiliations:** ^1^Department of Cardiology, Renmin Hospital of Wuhan University, Wuhan, Hubei 430060, China; ^2^Hubei Key Laboratory of Metabolic and Chronic Diseases, Wuhan, Hubei 430060, China; ^3^Department of Geriatrics, Renmin Hospital of Wuhan University, Wuhan, Hubei 430060, China

## Abstract

Myricetin (Myr) is a common plant-derived polyphenol and is well recognized for its multiple activities including antioxidant, anti-inflammation, anticancer, and antidiabetes. Our previous studies indicated that Myr protected mouse heart from lipopolysaccharide and streptozocin-induced injuries. However, it remained to be unclear whether Myr could prevent mouse heart from pressure overload-induced pathological hypertrophy. Wild type (WT) and cardiac Nrf2 knockdown (Nrf2-KD) mice were subjected to aortic banding (AB) surgery and then administered with Myr (200 mg/kg/d) for 6 weeks. Myr significantly alleviated AB-induced cardiac hypertrophy, fibrosis, and cardiac dysfunction in both WT and Nrf2-KD mice. Myr also inhibited phenylephrine- (PE-) induced neonatal rat cardiomyocyte (NRCM) hypertrophy and hypertrophic markers' expression *in vitro*. Mechanically, Myr markedly increased Nrf2 activity, decreased NF-*κ*B activity, and inhibited TAK1/p38/JNK1/2 MAPK signaling in WT mouse hearts. We further demonstrated that Myr could inhibit TAK1/p38/JNK1/2 signaling via inhibiting Traf6 ubiquitination and its interaction with TAK1 after Nrf2 knockdown in NRCM. These results strongly suggested that Myr could attenuate pressure overload-induced pathological hypertrophy *in vivo* and PE-induced NRCM hypertrophy via enhancing Nrf2 activity and inhibiting TAK1/P38/JNK1/2 phosphorylation by regulating Traf6 ubiquitination. Thus, Myr might be a potential strategy for therapy or adjuvant therapy for malignant cardiac hypertrophy.

## 1. Introduction

Pathological cardiac hypertrophy plays a central role in a variety of cardiovascular diseases and is an independent predictor for heart failure, arrhythmia, and sudden death [1, 2]. Many pathological stimuli, such as chronic hypertension, myocardial infarction, and aortic stenosis, could inevitably induce pathological hypertrophy [1, 2]. A series of malignant remodeling occurred in pathological hypertrophy, including enlarged cardiomyocyte, accelerated protein synthesis, cell death, aggravated fibrosis, dysregulated Ca^2+^-handling, disturbed mitochondrial function, and reactivated fetal gene expression [1, 2]. Multiple signaling pathways, such as mitogen-activated protein kinase (MAPK), NF-*κ*B, and Nrf2/HO-1, were dysregulated and were demonstrated to involve in promoting malignant remodeling. Targeting at these dysregulated signaling might find out strategies for protesting against pathological hypertrophy.

Myricetin (Myr) is a well-known polyphenol and possesses a similar molecular structure with kaempferol, quercetin, morin, and fisetin [3]. Scientific investigations had revealed that Myr possessed multiple pharmacological activities including antioxidant, antiphotoaging, anti-inflammation, antidiabetes, and anticancer [3]. Myr has also been demonstrated to protest against cardiovascular disease in both clinical and basic researches. Myr intake from daily diet was inversely associated with the incidence of cardiovascular diseases [4, 5]. In basic researches, Myr treatment protected isoproterenol- (ISO-) induced myocardial infarction via increasing superoxide dismutase (SOD) and catalase (CAT) [6]. Myr exerted a more pronounced capacity than quercetin for preventing STAT1 activation in ischemia/reperfusion [7] and consequently showed more cardiovascular benefits. Myr treatment could also promote coronary dilation via increasing intracellular cGMP without affecting cardiomyocyte systolic and diastolic function [8]. These investigations implied that Myr might be a potential drug for therapy or adjuvant therapy in various cardiovascular diseases, but the underling mechanisms remain to be further explored.

In our laboratory, we have demonstrated that Myr attenuated LPS-induced mouse heart injuries by inhibiting oxidative stress and NF-*κ*B/p65 activity [9]. We further demonstrated that the antioxidant activity of Myr might be associated with enhancing Nrf2/HO-1 pathway in diabetic mouse heart. However, Myr remained to inhibit NF-*κ*B activity after Nrf2 silence in neonatal rat cardiomyocytes (NRCMs) [10]. Thus, some mechanisms independent of Nrf2/HO-1 remain to be investigated in mouse heart. Also, none published studies have investigated the roles and mechanisms of Myr in pressure overload-induced pathological hypertrophy. In this study, we intended to investigate whether Myr could protect mouse heart from pressure overload-induced hypertrophy and clarify the underlying mechanisms.

## 2. Methods

### 2.1. Reagents

Myr (>99% purity) was purchased from Shanghai Winherb Medical Science Co., Ltd. (Shanghai, China). BCA protein assay kit was purchased from Pierce (Rockford, IL, USA). The following primary antibodies were used in this study: T-ERK (CST, 4695), p-JNK (T183/Y185) (CST, 4668P), T-JNK (CST, 9258S), T-TAK1 (CST, 4060), p-P38 (CST, 4511P), T-p38 (CST, 9212P), p-TAK1 (CST, 4508), p-P65 (s276) (BIOWORLD, BS4135), T-P65 (CST, 8242), GAPDH (CST, 2118), p-ERK1/2 (Thr202/Tyr 204) (CST, 4370P), Histone-3 (Abcam, ab5176), TRAF6 (Santa Cruz, sc-8409), ubiquitin (Proteintech, 10201-2-AP), Nrf2 (Proteintech, 16396-1-AP), 4-hydroxynonenal (Abcam, ab46545), SOD1 (Abcam, ab16831), and catalase (Proteintech, 19792-1-AP). Lip 6000 (C0528) and protein A+G Agarose were purchased from Beyotime (Jiangsu, China). Peroxidase-conjugated secondary antibodies were from Jackson ImmunoResearch Laboratories (1 : 10000); fetal calf serum was from HyClone (Waltham, MA, USA).

### 2.2. Animal Models

All experimental protocols used for animal experiments in this study were approved by the Animal Care and Use Committee of Renmin Hospital of Wuhan University. All surgery procedures were performed according to the National Institutes of Health (NIH) Guide for the Care and Use of Laboratory Animals.

#### 2.2.1. Generation of Cardiac-Specific Nrf2 Knockdown (Nrf2-KD) Mice

Nrf2 shRNA Lentiviral Particles (for mouse) were purchased from Santa Cruz Biotechnology (sc-37049-v). Intramyocardial injection of lentiviral particles was performed according to our previous study [11]. Briefly, mice were anaesthetized with pentobarbital sodium and mechanically ventilated. A horizontal skin incision was made at the 3-4 intercostal space. The heart was smoothly and gently “popped out.” Lentiviral particles were injected into the left ventricle with a syringe equipped with a 31-gauge needle. The sham group was injected with scram shRNA Lentiviral Particles. Three different injection points were selected around the left ventricle. After injection, mouse heart was immediately put back and then close muscle and suture the skin. All mice were allowed for shRNA expression for 2 weeks and then were prepared for following experiments.

#### 2.2.2. Pressure Overload-Induced Hypertrophy Model

Aortic banding (AB) surgery was performed to establish pathological hypertrophy models according to previous depict [11]. Briefly, male mice (C57/BL6, age 8-10 weeks and weight 23.5-27.5 g) were anaesthetized with pentobarbital sodium (50 mg/kg, Sigma) by intraperitoneal injection. After losing toe pinch reflex, open the left chest at the 2-3 intercostal space to expose the aorta with blunting dissection. The aorta was tied with a 27-gauge or 26-gauge needle using a 7-0 silk suture. After ligation, the needle was removed gently to cause aortic constriction. Sham mice went through a similar procedure without aorta ligation.

#### 2.2.3. Animal Groups and Myr Administration

After three days of AB surgery or sham operation, mice were allocated into different experimental groups randomly. In this study, two independent animal experiments were performed. Firstly, animals were allocated into four groups: sham+normal saline (NS) group (control group, CON), sham+Myr treatment group (Myr), AB+NS (AB) group, and AB+Myr (AB+M) group. Secondly, animals were allocated into scram shRNA+sham+vehicle (NS) (sham+scram+Veh), scram shRNA+sham+Myr (sham+scram+Myr), shRNA+AB+Veh (shRNA+Veh+AB), and shRNA+AB+Myr. Myr were dissolved in NS and were administrated by gastric needle for consecutive 6 weeks with a dose of 200 mg/kg/d, which have been performed according to our previous studies [9, 10]. The control groups were treated with NS.

### 2.3. Echocardiographic Analysis

Cardiac function was measured by echocardiography before mouse sacrifice. Mice were anesthetized by 1.5% isoflurane. Mylab 30CV (ESAOTE S. P. A) equipped with a 15 MHZ linear-array ultrasound transducer was used to assess the left ventricle (LV) dimension at the parasternal short axis. M-mode tracing was used to detect and calculate the following parameters: interventricular septal thickness (IVS) at diastole (IVSd), IVS at systole (IVSs), LV end-diastolic diameter (LVEDd), LV end-systolic diameter (LVEDs), LV end-diastolic posterior wall thickness (LVPWd), LV end-systolic posterior wall thickness (LVPWs), LV ejection fraction (LVEF), and fractional shortening (FS). End-diastole or systole was defined as the phase of the largest or smallest area of the LV, respectively.

### 2.4. Histology Analysis

After echocardiography measurement, mice were sacrificed by cervical dislocation. Heart weight (HW), lung weight (LW), and tibia length (TB) were recorded to calculate the HW/BW, LW/BW, and HW/TL. Then, mouse hearts were arrested in diastole in 10% KCl and fixed by 10% formalin for 12 h. After dehydration, hearts were embedded in paraffin and cut transversely close to the apex with 4-5 *μ*m thickness. Hematoxylin-eosin (HE) and picrosirius red (PSR) staining were performed to assess the cardiomyocyte cross-sectional area (CSA) and collagen volume in the LV. A digital image analysis system (Image-Pro Plus, version 6.0) was used for image capture and analysis.

### 2.5. Neonatal Rat Cardiomyocyte (NRCM) Culture and Treatment

Neonatal rat cardiomyocytes (NRCMs) were isolated from 1-3 day old Sprague-Dawley rats according to published protocol [11]. Briefly, NRCMs were isolated in D-hanks buffer containing 0.125% trypsin for repeated digestion with 15 min × 5 times. The harvested cells were resuspended in DMEM/F12 medium supplemented with 15% fetal bovine serum (FBS). A differential attachment method was used to remove cardiac fibroblasts from NRCMs, and then, NRCMs were seeded at a density of 2 × 10^5^ cells per well in 6-well plates for protein or mRNA extraction and 1 × 10^4^ cells per well in 24-well plates for immunofluorescence staining. NRCMs were incubated with 0.1 mmol/L bromodeoxyuridine (BrdU) in DMEM/F12 medium with 15% FBS for 36 h before the following experiments.

NRCMs were transfected with gene-specific siRNA for Nrf2 silence (SANTACRUZ, SC-156128) or plasmid for TAK1 overexpression using Lipo 6000 transfection reagent according to the manufacturer's instructions. After another 24 h incubation, NRCMs were cultured in serum-free DMEM/F12 for 12 h and then were stimulated with PE (50 *μ*M) or Myr (20 *μ*M). The treatment dosage of Myr was determined according to our previous publication [9].

Immunofluorescence staining was performed to evaluate NRCM hypertrophy. NRCMs were fixed with 4% paraformaldehyde solution for 10 min at room temperature and then washed with PBS for 3 min × 5 times. After permeabilization with 0.1% Triton X-100 in PBS for 15 min, NRCMs were stained with anti-cardiac Troponin T antibody (1 : 100) overnight. In the next day, the secondary antibody, Alexa Fluor 488 anti-mouse IgG, was used to label cTnT in green. The NRCMs were mounted on glass slides with SlowFade Gold antifade reagent with DAPI.

To demonstrate that Myr treatment could cause antioxidative stress via partly regulating Nrf2, NRCMs were transfected with specific siRNA for Nrf2 knockdown. After transfection of siRNA for 24 h, NRCMs were incubated with Myr or H_2_O_2_ for another 48 hours. And then, NRCMs were harvested for detecting the expression of Nrf2-regulated genes by RT-PCR and antioxidative enzymes by western blots.

### 2.6. Western Blot Analysis

Mouse heart tissue or NRCMs were lysed in RIPA buffer. To examine NF-*κ*B/P65 and Nrf2 nucleus translocation, nucleus protein was extracted by a commercial kit purchased from Jiancheng Bioengineering Institute (Nanjing, China). Protein concentration was determined by a BCA protein assay kit. 50 *μ*g protein was used for electrophoresis on 10% SDS-PAGE gels and transferred to a polyvinylidene fluoride membrane (Millipore). After blocking with 5% BSA for 1 h, the blots were incubated with corresponding primary antibodies overnight at 4°C. The peroxidase-conjugated secondary antibodies were used to incubate with the blots for 1 h in the next day. The blots were visualized using Bio-Rad ChemiDocTX XRS+. All expressions of proteins were normalized to corresponding GAPDH before relative quantitative calculation.

### 2.7. Quantitative Real-Time PCR (rt-PCR)

Total mRNA was extracted from snap-frozen heart tissue or NRCMs using the TRIzol reagent according to the manufacturer's instructions. Harvested mRNA was spectrophotometrically estimated by A260/A280 and A230/260 relying on the Smartspec Plus Spectrophotometer (Bio-Rad). Total mRNA (2 *μ*g/sample) was converted into cDNA using the Transcriptor First Strand cDNA Synthesis Kit. RT-PCR amplification of target genes in this study was performed using LightCycler 480 SYBR Green 1 Master Mix. All expression levels of genes were normalized to GAPDH before relative quantitative calculation. Primers used in this study are shown in Table 1.

### 2.8. Measured Reactive Oxygen Species

Reactive Oxygen Species (ROS) Assay Kit (Beyotime, Shanghai, China) was purchased for ROS measurement according to the manufacturer's instruction. Briefly, NRCMs were transfected with siRNA for 24 h and then were treated with or without Myr (20 *μ*M) overnight. NRCMs were incubated with 50 *μ*M dichlorofluorescein diacetate (DCFDA) at 37°C in the dark for 30 min. DCFDA in cells could be cleaved into nonfluorescent 2,7-dichlorofluorescin, which could be oxidized by intracellular ROS to product fluorescent dichlorofluorescein (DCF). NRCMs were then incubated with H_2_O_2_ (30 *μ*M) for 30 min in the dark. Finally, NRCMs were trypsinized and counted for fluorescence quantification in a fluorescence plate reader (BioTek) (*λ*_Ex_ = 488, *λ*_Em_ = 525).

### 2.9. HEK 293T Culture and Plasmid Transfections

Plasmid of Prk5-HA-Ubiquitin-K63 (K63-Ub) was obtained from addgene, which only expressed ubiquitin with K63, and other lysines were mutated to arginines [12]. The cDNA of TAK1 (NM 145331) was purchased from Vigene Biosciences (China) and cloned into pc-DNA 3.1 system (Invitrogen, USA) for TAK1 overexpression. HEK393T cells were purchased from China Center for Type Culture Collection (Wuhan, China) and prepared according to published protocol [13]. Briefly, HEK293T cells were seeded in 6-well plates with culture medium supplemented with 10% FBS. Cells were transfected with plasmid (2 *μ*g) by Lipo 6000™ transfection reagent when cell density reached the 60-70% area of the culture dish. After 24 h transfection, cells were treated with 50 *μ*M PE or 20 *μ*M Myr for another 30 min. Finally, the cells were washed twice with 4 ml ice-cold PBS and lysed on ice with 70 *μ*l lysis buffer in each well. Six-well plates were swirled on ice for 15 min, and the cell debris was collected with a cell scraper for following experiments.

### 2.10. Immunoprecipitation and Ubiquitination Assay

Protein lysate from NRCMs or HEK293T cells was immunoprecipitated with anti-Traf6 coupled to protein A+G Agarose (Beyotime, Shanghai, China). Briefly, protein concentration was determined by BCA assay. Sufficient primary monoclonal antibody of Traf6 was added into 200 *μ*g proteins and incubated at 4°C overnight with gentle rotation. The next day, the beads containing complexes were collected by centrifugation at 2500 rpm for 5 min. The containing complexes were washed and boiled in SDS-PAGE loading buffer. The immunoprecipitated proteins were electrophoresed on 10% SDS-PAGE gels and transferred to a polyvinylidene fluoride membrane (Millipore). Blots were incubated with anti-TAK1 and antiubiquitin to determine the Traf6 ubiquitination and interaction between Traf6 and TAK1.

### 2.11. Statistical Analysis

All data were presented with mean ± s.d. One-way analysis of variance (ANOVA) was used to compare means among groups followed by least significant difference (LSD, equal variances) or Tamhane' s T2 (none equal variances) tests. Image-Pro 6.0 was used for quantitative analysis of western blots and pictures. SPSS 19.0 was used for statistical analysis in this study. *p* < 0.05 was considered significant.

## 3. Results

### 3.1. Myr Treatment Attenuated Pathological Cardiac Hypertrophy and Fibrosis

**Figure 1:**
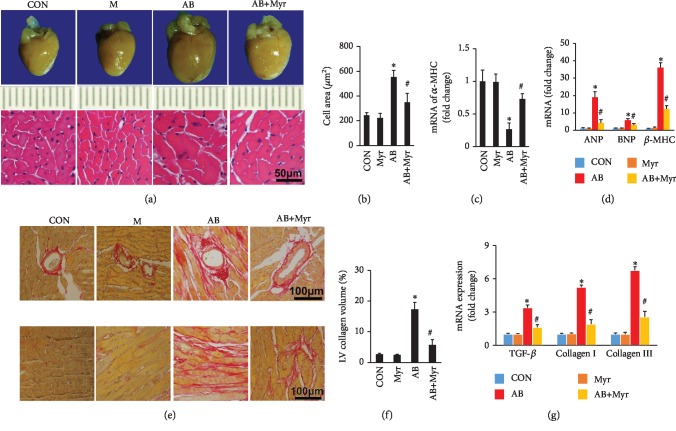
Myr treatment attenuated cardiac hypertrophy and fibrosis. (a) Gross image of the heart; (b) calculated cardiomyocyte across the area of HE staining; (c) Myr prevented the downregulation of *α*-MHC; (d) Myr inhibited the expression of ANP, BNP, and *β*-MHC; (e) Myr inhibited interstitial and perivascular fibrosis in mouse heart; (f) calculated LV collagen volume of PSR staining; (g) Myr inhibited the expression of fibrosis-associated markers, *n* ≥ 6 for staining experiments, *n* = 4 for mRNA determination, ^∗^*p* < 0.05 versus the CON group or the Myr group, ^#^*p* < 0.05 versus the AB+Myr group.

As presented in Table 2, pressure overload induced obvious cardiac hypertrophy evidenced by increased HW, HW/BW, and HW/TB ratios compared with CON and Myr groups. However, Myr treatment significantly alleviated cardiac hypertrophy evidenced by decreased HW, HW/BW, and HW/TB. HE staining showed significant enlargement of cardiomyocyte (Figures 1(a) and 1(b)) and overproduction of ANP and BNP after AB surgery (Figure 1(c)). Besides, the adult *α*-MHC was downregulated in mouse heart whereas the fetal *β*-MHC was significantly upregulated compared with CON and Myr groups (Figures 1(c) and 1(d)). Myr treatment significantly blunted these pathological changes induced by pressure overload (Figures 1(a)–1(d)).

Cardiac fibrosis is an integrate process in the development of pathological cardiac hypertrophy. In this study, AB induced significant interstitial and perivascular fibrosis compared with CON and Myr groups (Figures 1(e) and 1(f)); however, Myr treatment significantly attenuated cardiac fibrosis and inhibited fibrosis-associated markers' expression compared with the AB group (Figures 1(e)–1(g)). Persistent cardiac hypertrophy and fibrosis directly contributed to cardiac dysfunction and heart failure. After 6 weeks of pressure overload, IVSd, LVEDd, LVEPWd, and LVEDs were significantly increased while EF and FS were markedly decreased compared with the CON group or the Myr group (Table 3). Myr treatment significantly improved cardiac function evidenced by decreased IVSd, LVEDd, LVEPWd, and LVEDs, as well as increased EF and FS compared with the AB group (Table 3).

### 3.2. Myr Enhanced the Nrf2/HO-1 and Inhibited MAPK/NF-*κ*B

**Figure 2:**
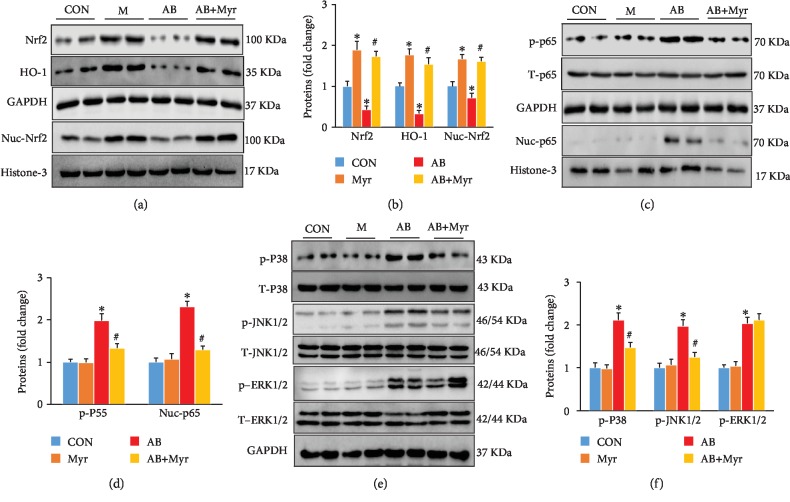
Protein levels of signaling pathways after Myr treatment. (a, b) Myr inhibited the Nrf2/HO-1 pathway; (c, d) Myr inhibited the phosphorylation and nuclei translocation of p65; (d, e) Myr inhibited the phosphorylation of p38 and JNK1.2 MAPK kinase. *n* = 6, ^∗^*p* < 0.05 versus the CON group or the Myr group, ^#^*p* < 0.05 versus the AB+Myr group.

In our previous study, we have showed that Myr treatment could significantly enhance the Nrf2/HO-1 pathway and block NF-*κ*B nuclei translocation [9, 10]. In this study, we also detected the enhanced expression and nuclei translocation of Nrf2 resulted in the activation of the Nrf2/HO-1 pathway (Figures 2(a) and 2(b)), which was downregulated under chronic pressure overload (Figures 2(a) and 2(b)). In addition, chronic pressure overload induced hyperphosphorylation and translocation of NF-*κ*B/p65 (Figures 2(c) and 2(d)), which was also prevented by Myr treatment (Figures 2(c) and 2(d)). Moreover, MAPK signaling (JNK1/2, P38, and ERK1/2) was significantly activated under pressure overload (Figures 2(e) and 2(f)); Myr treatment significantly blocked JNK1/2 and P38 overactivation compared with the AB group (Figures 2(e) and 2(f)), but no significant difference of ERK1/2 phosphorylation was found between the AB group and the AB+Myr group (Figures 2(e) and 2(f)).

### 3.3. Myr Partly Alleviated Pathological Cardiac Hypertrophy after Nrf2 Knockdown

**Figure 3:**
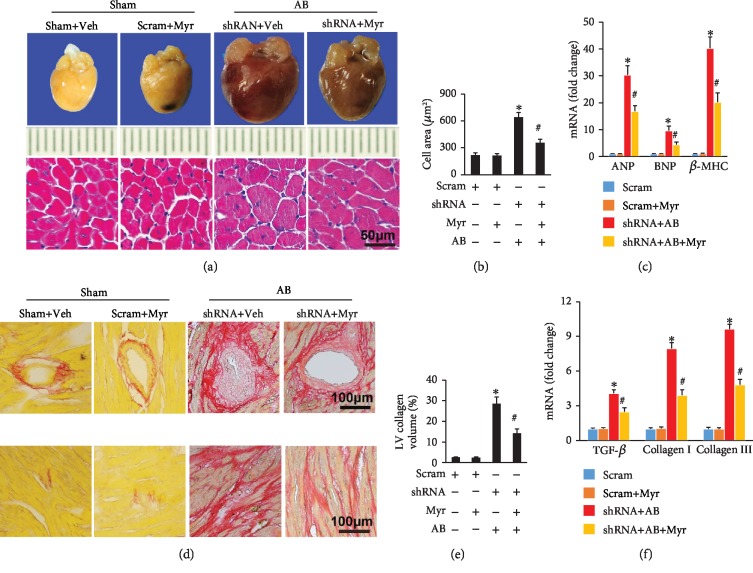
Myr inhibited cardiac hypertrophy and fibrosis after Nrf2 knockdown. (a) Myr inhibited mouse heart and cardiomyocyte hypertrophy; (b) calculated cardiomyocyte across the area of HE staining; (c) Myr inhibited the expression of ANP, BNP, and *β*-MHC; (d) Myr inhibited interstitial and perivascular fibrosis in mouse heart; (e) calculated LV collagen volume of PSR staining; (f) Myr inhibited the expression of fibrosis-associated markers, *n* ≥ 6 for staining experiments, *n* = 4 for mRNA determination, ^∗^*p* < 0.05 versus the scram group or the scram+Myr group, ^#^*p* < 0.05 versus the shRNA+AB group.

Based on previous result that enhanced Nrf2 expression could contribute to MAPK and NF-*κ*B pathway inhibition, we investigated here whether Myr could still prevent cardiac hypertrophy after Nrf2 knockdown (Nrf2-KD). To address this question, mouse hearts were firstly injected with lentivirus-wrapped shRNA or scram RNA for Nrf2-KD or negative control, respectively. After lentivirus injection for 3 weeks, mice were subjected to AB surgery and then treated with Myr for another 6 weeks. Unexpectedly, Myr treatment still obviously reversed pressure overload-induced cardiac hypertrophy (Table 4) and dysfunction (Table 5) after Nrf2-KD, as evidenced by decreased HW/BW, LW/BW, HW/TL, IVSd LVEDd, LVEPWd, LVEDs, and LEPWs compared with the shRNA+AB group (Tables 4 and 5). The EF and FS were also significantly improved in the shRNA+AB+Myr group compared to the shRNA+AB group (Table 5). HE and PSR staining presented consistent results that Myr treatment significantly attenuated cardiomyocyte hypertrophy and fibrosis compared with the shRNA+AB group (Figures 3(a), 3(b), 3(d), and 3(e)). Similarly, hypertrophic-associated markers (ANP, BNP, and *β*-MHC) and fibrosis-associated markers (TGF-*β* and collagen I/III) were also significantly inhibited by Myr treatment compared with the shRNA+AB group (Figures 3(c) and 3(e)). Obviously, Myr remained to be partly beneficial for protecting against pressure overload-induced cardiac hypertrophy after Nrf2-KD.

### 3.4. Myr Treatment Inhibited the TAK1/MAPK Pathway Independent of Nrf2

**Figure 4:**
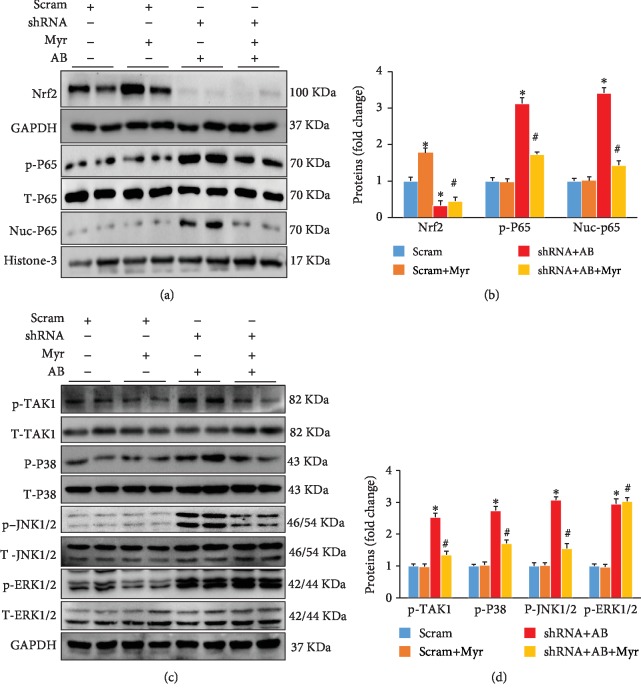
Determination of signaling pathways after Nrf2 knockdown in mouse heart. (a, b) Myr treatment inhibited the phosphorylation and nuclei translocation of p65; (c, d) Myr treatment inhibited the phosphorylation of TAK1/p38/JNK1/2 after Nrf2 knockdown, *n* = 6, ^∗^*p* < 0.05 versus the scram group or the scram+Myr group, ^#^*p* < 0.05 versus the shRNA+AB group.

Nrf2 was significantly downregulated in mouse heart after shRNA injection compared with the scram group or the scram+Myr group (Figures 4(a) and 4(b)). Myr treatment remained to inhibit NF-*κ*B/p65 hyperphosphorylation and Nrf2 nucleus translocation after Nrf2 knockdown (Figures 4(a) and 4(b)). Meanwhile, Myr treatment could inhibit pressure overload-induced JNK and p38 hyperphosphorylation (Figures 4(c) and 4(d)) but showed none significant effects for ERK1/2 hyperphosphorylation (Figures 4(c) and 4(d)). Further investigations indicated that Myr treatment markedly blocked TAK1 excessive phosphorylation, which was the common upstream regulator of p-JNK and p-p38 (Figures 4(c) and 4(d)). According to these results, it was reasonable to deduce that Myr treatment prevented cardiac hypertrophy at least partly through inhibiting the TAK1/MAPK pathway.

### 3.5. Myr Prevented PE-Induced NRCM Hypertrophy and H_2_O_2_-Induced Oxidative Stress

**Figure 5:**
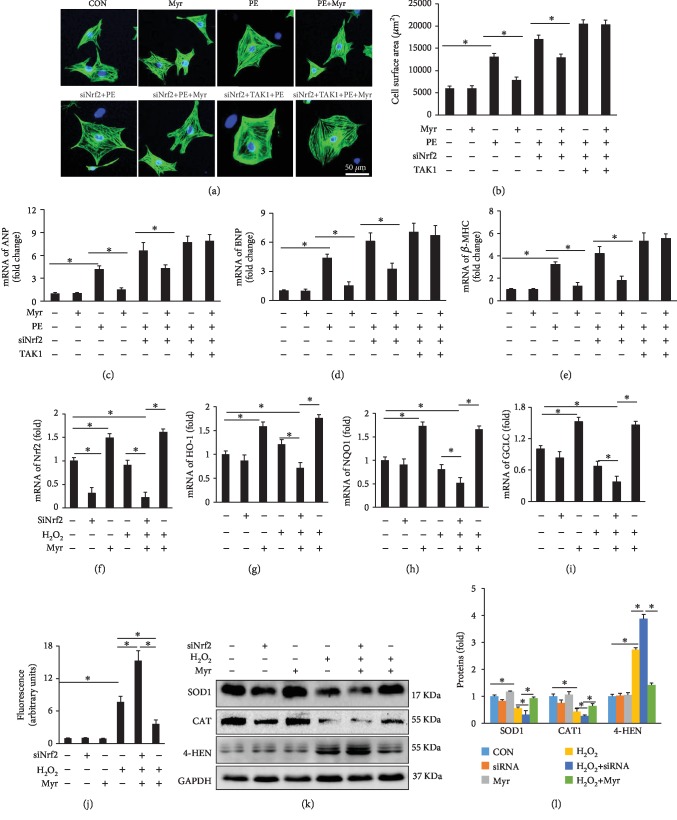
Myr prevented NRCM hypertrophy *in vitro*. (a) Immunofluorescence (IF) staining of NRCM in different groups; (b) calculated cell surface area (>100 cells per group); (c–e) mRNA expression levels of ANP, BNP, and *β*-MHC, respectively; Nrf2 knockdown (f) and its downstream genes including HO-1 (g), NQQ-1 (h), and GCLC (i), respectively. All mRNA expressions of target genes were normalized to GAPDH; (j) DCF fluorescence was quantified to present the reactive oxygen species after different treatments indicated in the pictures; (k) representative western blots for SOD1, CAT, and 4-HEN after different treatments indicated in the pictures; (l) quantified SOD1, CAT1, and 4-HEN after normalized to GAPDH. Cellular experiments were repeated three times independently. ^∗^*p* < 0.05 versus the group indicated in the picture.

In *in vitro* experiment, PE treatment induced significant NRCM hypertrophy, which could be markedly inhibited by Myr treatment (Figures 5(a) and 5(b)). After Nrf2 knockdown, Myr could also partly prevent NRCM from PE-induced hypertrophy (Figures 5(a) and 5(b)). However, after Nrf2 knockdown and TAK1 overexpression in NRCM, Myr could not protect NRCMs from PE-induced hypertrophy (Figures 5(a) and 5(b)). RT-PCR was also performed to examine hypertrophy-associated biomarkers in vitro. Myr treatment could significantly prevent overexpression of ANP, BNP, and *β*-MHC in both the PE group and the PE+siNrf2 group (Figures 5(c) and 5(d)); however, after overexpression of TAK1 in Nrf2 knockdown cells, Myr could no longer attenuate PE-induced hypertrophic biomarkers' expression (Figure 5(c) and 5(d)).

To further demonstrate that Myr might possess antioxidant stress via regulating Nrf2, NRCMs were treated with H_2_O_2_ or Myr with or without Nrf2 knockdown. Nrf2 downstream genes including HO-1, NQO1, and GCLC were significantly downregulated in the siNrf2+H_2_O_2_+Myr group compared to the H_2_O_2_ treatment group (Figures 5(f)–5(i)). Myr treatment could not upregulate the expression of HO-1, NQO1, and GCLC compared to control groups after Nrf2 knockdown (Figures 5(f)–5(i)); however, Myr treatment could significantly upregulate these Nrf2-associated downstream genes in none Nrf2 knockdown NRCMs (Figures 5(f)–5(i)). DCF measurement demonstrated that Myr could attenuate H_2_O_2_-induced ROS accumulation in Nrf2 none knockdown NRCMs not in Nrf2 knockdown NRCMs (Figure 5(j)). H_2_O_2_ treatment 24 h induced significant upregulation of 4-HEN and downregulation of SOD1 and CAT compared to the control group (Figures 5(k) and 5(l)). Importantly, Myr treatment could inhibit 4-HEN expression and restore SOD1 and CAT expressions (Figures 5(k) and 5(l)); however, Myr could not inhibit 4-HEN expression and restore SOD1 and CAT expressions in Nrf2 knockdown NRCMs (Figures 5(k) and 5(l)). These results suggested that Myr-mediated Nrf2 expression prevented H_2_O_2_-induced oxidative stress in NRCMs.

### 3.6. Myr Prevented TRAF6/TAK1 Interaction via Regulating Traf6 Ubiquitin

**Figure 6:**
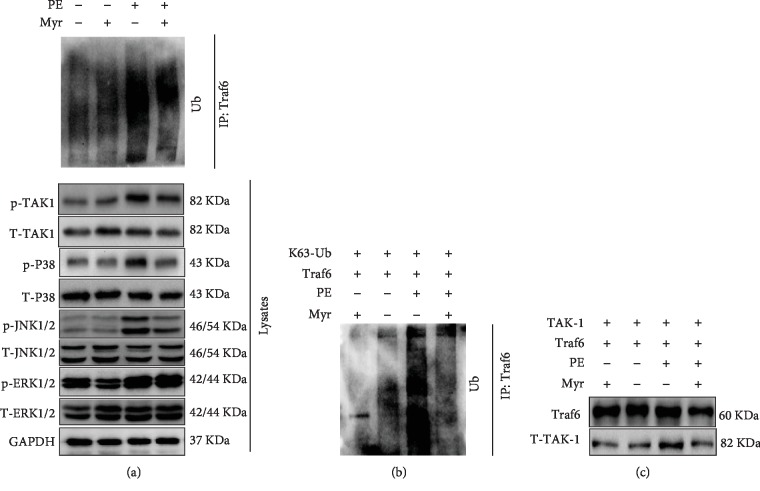
Myr treatment prevented Traf6 ubiquitination *in vitro*. (a) Myr (20 *μ*M, preincubation with NRCM for 1 h) treatment prevented PE (50 *μ*M, 30 min) induced TRAF6 ubiquitination and the phosphorylation of p38 and JNK1/2; (b) Myr treatment inhibited K63-Ub-induced TRAF6 ubiquitination in HEK293 cell; (c) Myr inhibited the interaction between Traf6 and TAK1, and TAK1phosphorylation. All experiments were repeated three times independently.

Finally, we intended to investigate the exact mechanism from which Myr regulated the TAK1/MAPK pathway. Previous studies suggested that polyphenols could regulate protein ubiquitination [14, 15]. Here, we investigated whether Myr could regulate Traf6 ubiquitination. PE treatment induced Traf6 ubiquitination, while Myr treatment significantly inhibited Traf6 ubiquitination in NRCM (Figure 6(a)). It was also observed that Myr treatment prevented hyperphosphorylation of TAK1, P38 and JNK1/2 but not ERK1/2 phosphorylation in PE-treated NRCMs (Figure 6(a)). To further investigate the ubiquitination regulation manner, K63-only ubiquitin (K63-Ub) and Traf6 were cloned into plasmid and then were cotransfected into HEK293 cell. After PE treatment, Traf6 was obviously ubiquitinated by K63 ubiquitin, and Myr treatment significantly prevented this ubiquitin (Figure 6(b)). Finally, TAK-1 and Traf6 were cotransfected into HEK293 cell (Figure 6(c)). PE treatment caused significant interaction between TAK-1 and Traf6 (Figure 6(c)), but Myr treatment obviously disturbed the interaction between TAK1 and Traf6 (Figure 6(c)).

## 4. Discussion

This study demonstrated for the first time that Myr protected mouse heart and NRCM from pressure overload or PE-induced hypertrophy, respectively. Hypertrophic stimuli caused Nrf2/HO-1 downregulation and TRAF6 unregulation and its ubiquitination. Ubiquitinated TRAF6 could interact with TAK1 promoting TAK1/JNK1/2/P38 phosphorylation. Myr treatment significantly inhibited the TRAF6/TAK1/MAPK pathway and restored Nrf2/HO-1 activity. Moreover, Traf6/TAK1/MAPK cascade inhibition was independent of Nrf2/HO-1 activity. In addition, Myr treatment prevented Traf6 expression and ubiquitination and also prevented the TRAF6-TAK1 interaction.

Previous investigations have presented that Myr was a powerful antioxidant in various cells and diseases [3]. Myr could increase the activity and protein expression of many antioxidases including SOD, GSH/GSSG ratio, catalase, and glutathione. These antioxidases were markedly downregulated in ischemia/reperfusion-induced myocardial injury and deoxycorticosterone acetate- (DOCA-) salt-hypertensive rats [16, 17]. Myr reacted with oxygen-centered galvinoxyl radicals 28 times faster than vitamin E [18]. Gene microarray analysis in HepG2 cells suggested that Myr might involve in promoting Nrf2-mediated antioxidant response element (ARE) activation for exerting its antioxidant roles [19]. Further investigations revealed that Myr treatment prevented Nrf2 ubiquitination and protein turnover, promoting Nrf2 expression and kelch-like erythroid cell-derived protein with CNC homology-associated protein 1 modification [19]. Our previous study also demonstrated that Myr treatment attenuated diabetes-associated cardiomyocyte hypertrophy, apoptosis, and interstitial fibrosis via restoring Nrf2/HO-1 pathway activity [10]. Nrf2 has been demonstrated to play important roles in regulating cardiac pathophysiology. Published data have showed that deficiency or downregulation of Nrf2 exacerbated pathological cardiac hypertrophy [20, 21], while stimulating Nrf2 expression by genetic or drug treatment strategies could significantly attenuate pathological remodeling [22, 23]. Taken together these evidences, Myr-mediated Nrf2 regulation was undoubtedly one of the most important mechanisms for preventing pathological cardiac remodeling. Some clinical investigations have figured out that Myr intake was inversely associated with the morbidity of myocardial infarction or coronary heart disease, but the underlying mechanisms remained to be unclear [4, 24].

Our previous investigation showed that Myr treatment could improve LPS-induced cardiac injury by inhibiting I*κ*B/NF-*κ*B (p65) signaling and inflammatory cytokine secretion [9]. In a high glucose-induced NRCM injury model, we further demonstrated that Myr-mediated I*κ*B/NF-*κ*B (p65) inhibition was independent of Nrf2 enhancement [10]. In this study, we observed that Myr treatment inhibited p65 phosphorylation and nuclei translocation in both wild type and Nrf2-knockdown mouse hearts. These studies intensively displayed that Myr possessed potent anti-inflammatory activity. In lipoteichoic acid- (LTA-) treated human gingival fibroblasts (HGFs), Myr treatment depressed p38 and ERK1/2 activation and also inhibited I*κ*B*α* degradation [23, 25]. In IL-1*β*-stimulated SW982 synovial cells, Myr significantly reduced JNK and p38 phosphorylation [26]. These results implied that Myr-mediated inflammation reduction might be associated with MAPK signaling regulation. Based on these previous studies, this study detected MAPK signaling and figured out that Myr treatment significantly inhibited p38 and JNK1/2 phosphorylation but showed none significant effects for ERK1/2 phosphorylation.

TAK1 is an upstream regulatory protein of p38 and JNK1/2 MAPK [27, 28]. TAK1 phosphorylation could contribute to p38 and JNK1/2 MAPK hyperphosphorylation resulted in aggravated pathological cardiac hypertrophy [27, 28]. In this study, Myr treatment significantly depressed TAK1 phosphorylation after Nrf2 knockdown in *in vivo* experiment. Accordingly, we deduced that Myr alleviated cardiac hypertrophy through inhibiting TAK1 activation, which could be regulated by TRAF6 ubiquitination. Ji et al. [29] demonstrated that Traf6 autoubiquitination promoted the interaction of TRAF6 and TAK1 in the process of pressure overload-induced cardiac hypertrophy, and Traf6 deletion or inhibiting Traf6 ubiquitin effectively attenuated pressure overload-induced cardiac hypertrophy [29]. In previous studies, polyphenols have been demonstrated to suppress NF-*κ*B activation through decreasing Traf6 ubiquitination in Hela-T6RZC stable cells and to disrupt the polyubiquitin synthesis in *in vitro* kinase assay system [15]. Resveratrol, another well-known polyphenolic compound, has also been demonstrated to inhibit LPS-induced p38 and JNK1/2 activation via diminishing TRAF6 ubiquitination [14].

Based on these studies, we investigated whether Myr would show similar function with other polyphenolic compounds to regulate Traf6 ubiquitination. PE induced Traf6 ubiquitination in NRCM accompanied with increased phosphorylation of TAK1, p38, JNK1/2, and ERK1/2. Myr treatment significantly inhibited the TRAF6 expression and its ubiquitination and decreased the activity of TAK1, p38, and JNK1/2. Previous studies have indicated that the TRAF6-associated ubiquitination activation was largely determined by a site-specific nondegradative Lys-63-linked autoubiquitination (K63-Ub) [29–31]. So, K63-Ub and TRAF6 plasmids were cotransfected into HEK-293-cells. PE treatment obviously promoted TRAF6 ubiquitination, which was significantly inhibited by Myr treatment. TRAF6 autoubiquitination promoted TRAF6 and TAK1 interaction. TRAF6 and TAK1 plasmids were cotransfected into HEK-293 cells to detect the TRAF6 and TAK1 interaction. Our results showed that PE treatment significantly promoted TRAF6 and TAK1 interaction, which could be markedly blocked by Myr treatment.

Taken together, Myr possessed potent capacity to restore Nrf2/HO-1 activity and to inhibit TAK1/p38/JNK1/2 MAPK signaling via inhibiting TRAF6 autoubiquitination. Thus, Myr might be a potential drug with multiple targets for therapy or adjunct therapy of pathological cardiac hypertrophy. However, some questions remain to be addressed in following studies. For example, a previous study suggested that Myr promoted Nrf2 expression and nuclear translocation via regulating KEAP1 ubiquitination, but this study presented that Myr regulated TRAF6 autoubiquitination. How does Myr regulate the TRAF6 and KEAP ubiquitination at the same time? What is the exact mechanism of ubiquitination regulation?

## Figures and Tables

**Table 1:** Primers used for RT-PCR.

Gene symbol	Genus	Sense-forward primer	Antisense-reverse primer
ANP	Mouse	ACCTGCTAGACCACCTGGAG	CCTTGGCTGTTATCTTCGGTACCGG
BNP	Mouse	GAGGTCACTCCTATCCTCTGG	GCCATTTCCTCCGACTTTTCTC
*α*-MHC	Mouse	GTCCAAGTTCCGCAAGGT	AGGGTCTGCTGGAGAGGTTA
*β*-MHC	Mouse	CCGAGTCCCAGGTCAACAA	CTTCACGGGCACCCTTGGA
TGF-*β*	Mouse	TGCGCTTGCAGAGATTAAAA	CGTCAAAAGACAGCCACTCA
Collagen I	Mouse	AGGCTTCAGTGGTTTGGATG	CACCAACAGCACCATCGTTA
Collagen III	Mouse	CCCAACCCAGAGATCCCATT	GAAGCACAGGAGCAGGTGTAGA
GAPDH	Mouse	ACTCCACTCACGGCAAATTC	TCTCCATGGTGGTGAAGACA
ANP	Rat	AAAGCAAACTGAGGGCTCTGCTCG	TTCGGTACCGGAAGCTGTTGCA
BNP	Rat	TTCCTTAATCTGTCGCCGCTGG	CAGCAGCTTCTGCATCGTGGAT
*β*-MHC	Rat	TCTGGACAGCTCCCCATTCT	CAAGGCTAACCTGGAGAAGATG
GAPHD	Rat	GACATGCCGCCTGGAGAAAC	AGCCCAGGATGCCCTTTAGT

**Table 2:** Effects of myricetin on mouse cardiac hypertrophy.

	CON	Myr	AB	AB+Myr
*N*	12	12	16	16
BW	28.89 ± 1.04	28.17 ± 0.0.97	28.96 ± 1.35	28.52 ± 1.37
HW	122.5 ± 5	120.25 ± 5.08	187.69±14.78^∗∗^	160.56 ± 20.33^##^
LW	132.08 ± 7.91	135.33 ± 8.59	162.25±15.04^∗∗^	148.56 ± 9.8^#^
TB	19.04 ± 0.5	18.92 ± 0.51	19.19 ± 0.36	19.22 ± 0.36
HW/BW	4.25 ± 0.28	4.27 ± 0.13	6.49±0.58^∗∗^	5.85 ± 0.83^##^
LW/BW	4.58 ± 0.36	4.81 ± 0.31	5.61±0.49^∗∗^	5.18 ± 0.39^#^
HW/TB	6.44 ± 0.37	6.36 ± 0.38	9.78±0.79^∗∗^	8.35 ± 1.20^##^

*N*: number; BW: body weight; HW: heart weight; LW: lung weight; TB: tibia length, ^∗^*p* < 0.05 versus the CON group or the Myr group, ^#^*p* < 0.05 versus the AB+Myr group.

**Table 3:** Effects of myricetin on echocardiographic parameters.

	CON	Myr	AB	AB+Myr
*N*	10	10	10	10
HR (bpm)	496 ± 15	487 ± 27	492 ± 33	478 ± 34
IVSd (mm)	0.79 ± 0.05	0.78 ± 0.05	0.86 ± 0.03^∗^	0.81 ± 0.06^#^
LVEDd (mm)	3.88 ± 0.23	3.73 ± 0.14	4.56 ± 0.40^∗^	3.97 ± 0.33^#^
LVEPWd (mm)	0.77 ± 0.05	0.79 ± 0.05	0.89 ± 0.05^∗^	0.83 ± 0.05^#^
IVDs (mm)	1.12 ± 0.04	1.15 ± 0.08	1.2 ± 0.07	1.19 ± 0.12
LVEDs (mm)	2.05 ± 0.18	1.98 ± 0.09	3.1 ± 0.34^∗^	2.4 ± 0.49^#^
LVEPWs (mm)	1.13 ± 0.05	1.2 ± 0.06	1.16 ± 0.05	1.16 ± 0.12
EF (%)	81.5 ± 1.87	80.83 ± 2.4	63.08 ± 2.71^∗^	71.45 ± 3^#^
FS (%)	45 ± 4.38	46.77 ± 2.15	31.67 ± 2.42^∗^	37.8 ± 2.54^#^

HR: heart rate; IVSd: interventricular septal thickness (IVS) at diastole; IVSs: IVS at systole; LVEDd: LV end-diastolic diameter; LVEDs: LV end-systolic diameter; LVPWd: LV end-diastolic posterior wall thickness; LVPWs: LV end-systolic posterior wall thickness; LVEF: LV ejection fraction; FS: fractional shortening. ^∗^*p* < 0.05 versus the CON group or the Myr group, ^#^*p* < 0.05 versus the AB+Myr group.

**Table 4:** Effects of myricetin on cardiac hypertrophy after Nrf2 knockdown.

	Scram	Scram+M	shRAN+AB	shRNA+AB+M
*N*	12	12	13	13
BW	25.85 ± 0.62	25.77 ± 0.72	24.76 ± 0.48	25.01 ± 0.58
HW	114.42 ± 5.2	119 ± 6.84	214.21 ± 21.9^∗^	185.67 ± 17.97^#^
LW	136.63 ± 11.55	139.84 ± 13.94	212.36 ± 28.04^∗^	177.75 ± 23.53^#^
TB	19.08 ± 0.36	19.2 ± 0.33	19.29 ± 0.25	19.17 ± 0.33
HW/BW	4.42 ± 0.24	4.61 ± 0.23	8.66 ± 0.97^∗^	7.42 ± 0.70^#^
LW/BW	5.29 ± 0.52	5.42 ± 0.56	8.59 ± 1.22^∗^	7.1 ± 0.90^#^
HW/TB	6.0 ± 0.32	6.1 ± 0.3	11.11 ± 1.19^∗^	9.69 ± 0.95^#^

*N*: number; BW: body weight; HW: heart weight; LW: lung weight; TB: tibia length; ^∗^*p* < 0.05 versus the scram group or the scram+Myr group, ^#^*p* < 0.05 versus the shRNA+AB group.

**Table 5:** Effects of myricetin on echocardiographic parameters after Nrf2 knockdown.

	Scram	Scram+Myr	shRNA+AB	shRNA+AB+Myr
*N*	10	10	10	10
HR (bpm)	487 ± 15	496 ± 15	491 ± 14	486 ± 14
IVSd (mm)	0.79 ± 0.05	0.78 ± 0.04	0.88 ± 0.02^∗^	0.80 ± 0.04^#^
LVEDd (mm)	3.88 ± 0.15	3.75 ± 0.11	5.08 ± 0.35^∗^	4.58 ± 0.16^#^
LVEPWd (mm)	0.78 ± 0.03	0.77 ± 0.04	0.91 ± 0.04^∗^	0.81 ± 0.07^#^
IVDs (mm)	1.13 ± 0.05	1.15 ± 0.05	1.15 ± 0.05	1.11 ± 0.13
LVEDs (mm)	2.23 ± 0.18	2.18 ± 0.15	4.13 ± 0.32^∗^	3.43 ± 0.14^#^
LVEPWs (mm)	1.12 ± 0.08	1.1 ± 0.09	0.94 ± 0.12^∗^	1.06 ± 0.15^#^
EF (%)	78.83 ± 4.54	78.5 ± 3.21	43.75 ± 5.9^∗^	53.63 ± 3.8^#^
FS (%)	42.67 ± 3.83	41.83 ± 2.93	19.25 ± 2.25^∗^	25.31 ± 2.97^#^

HR: heart rate; IVSd: interventricular septal thickness (IVS) at diastole; IVSs: IVS at systole; LVEDd: LV end-diastolic diameter; LVEDs: LV end-systolic diameter; LVPWd: LV end-diastolic posterior wall thickness; LVPWs: LV end-systolic posterior wall thickness; LVEF: LV ejection fraction; FS: fractional shortening. ^∗^*p* < 0.05 versus the scram group or the scram+Myr group, ^#^*p* < 0.05 versus the shRNA+AB group.

## Data Availability

If someone or any research requests data or any details of the experiment about this article, please contact the first author (Hai-han Liao, email address: liaohaihan@whu.edu.cn or Nan Zhang, email address: zhangnan0609@whu.edu.cn) or corresponding author (Qi-zhu Tang, email address: qztang@whu.edu.cn).

## References

[1] Nakamura M., Sadoshima J. (2018). Mechanisms of physiological and pathological cardiac hypertrophy. *Nature Reviews Cardiology*.

[2] Shimizu I., Minamino T. (2016). Physiological and pathological cardiac hypertrophy. *Journal of Molecular and Cellular Cardiology*.

[3] Semwal D., Semwal R., Combrinck S., Viljoen A. (2016). Myricetin: a dietary molecule with diverse biological activities. *Nutrients*.

[4] Geleijnse J. M., Launer L. J., Van der Kuip D. A., Hofman A., Witteman J. C. (2002). Inverse association of tea and flavonoid intakes with incident myocardial infarction: the Rotterdam Study. *The American Journal of Clinical Nutrition*.

[5] Hertog M. G., Feskens E. J., Hollman P. C., Katan M. B., Kromhout D. (1993). Dietary antioxidant flavonoids and risk of coronary heart disease: the Zutphen Elderly Study. *The Lancet*.

[6] Tiwari R., Mohan M., Kasture S., Maxia A., Ballero M. (2009). Cardioprotective potential of myricetin in isoproterenol-induced myocardial infarction in Wistar rats. *Phytotherapy Research*.

[7] Scarabelli T. M., Mariotto S., Abdel-Azeim S. (2009). Targeting STAT1 by myricetin and delphinidin provides efficient protection of the heart from ischemia/reperfusion-induced injury. *FEBS Letters*.

[8] Angelone T., Pasqua T., Di Majo D. (2011). Distinct signalling mechanisms are involved in the dissimilar myocardial and coronary effects elicited by quercetin and myricetin, two red wine flavonols. *Nutrition, Metabolism, and Cardiovascular Diseases*.

[9] Zhang N., Feng H., Liao H. H. (2018). Myricetin attenuated LPS induced cardiac injury in vivo and in vitro. *Phytotherapy Research*.

[10] Liao H.-h., Zhu J.-x., Feng H. (2017). Myricetin Possesses Potential Protective Effects on Diabetic Cardiomyopathy through Inhibiting I *κ* B *α* /NF *κ* B and Enhancing Nrf2/HO-1. *Oxidative Medicine and Cellular Longevity*.

[11] Yuan Y., Yan L., Wu Q. Q. (2016). Mnk1 (mitogen-activated protein kinase-interacting kinase 1) deficiency aggravates cardiac remodeling in mice. *Hypertension*.

[12] Lim K. L., Chew K. C., Tan J. M. (2005). Parkin mediates nonclassical, proteasomal-independent ubiquitination of synphilin-1: implications for Lewy body formation. *The Journal of Neuroscience*.

[13] Gupta A., Maccario H., Kriplani N., Leslie N. R. (2016). In cell and in vitro assays to measure PTEN ubiquitination. *Methods in Molecular Biology*.

[14] Jakus P. B., Kalman N., Antus C. (2013). TRAF6 is functional in inhibition of TLR4-mediated NF-*κ*B activation by resveratrol. *The Journal of Nutritional Biochemistry*.

[15] Wang K., Sawaya A. C. H. F., Hu L. (2015). Polyphenol-rich propolis extracts from China and Brazil exert anti- inflammatory effects by modulating ubiquitination of TRAF6 during the activation of NF-*κ*B. *Journal of Functional Foods*.

[16] Borde P., Mohan M., Kasture S. (2011). Effect of myricetin on deoxycorticosterone acetate (DOCA)-salt-hypertensive rats. *Natural Product Research*.

[17] Qiu Y., Cong N., Liang M., Wang Y., Wang J. (2017). Systems pharmacology dissection of the protective effect of myricetin against acute ischemia/reperfusion-induced myocardial injury in isolated rat heart. *Cardiovascular Toxicology*.

[18] Bennett C. J., Caldwell S. T., McPhail D. B., Morrice P. C., Duthie G. G., Hartley R. C. (2004). Potential therapeutic antioxidants that combine the radical scavenging ability of myricetin and the lipophilic chain of vitamin E to effectively inhibit microsomal lipid peroxidation. *Bioorganic & Medicinal Chemistry*.

[19] Qin S., Chen J., Tanigawa S., Hou D. X. (2013). Microarray and pathway analysis highlight Nrf2/ARE-mediated expression profiling by polyphenolic myricetin. *Molecular Nutrition & Food Research*.

[20] Erkens R., Kramer C. M., Lückstädt W. (2015). Left ventricular diastolic dysfunction in Nrf2 knock out mice is associated with cardiac hypertrophy, decreased expression of SERCA2a, and preserved endothelial function. *Free Radical Biology & Medicine*.

[21] Tian C., Gao L., Zimmerman M. C., Zucker I. H. (2018). Myocardial infarction-induced microRNA-enriched exosomes contribute to cardiac Nrf2 dysregulation in chronic heart failure. *American Journal of Physiology. Heart and Circulatory Physiology*.

[22] Smyrnias I., Zhang X., Zhang M. (2015). Nicotinamide adenine dinucleotide phosphate oxidase-4-dependent upregulation of nuclear factor erythroid-derived 2-like 2 protects the heart during chronic pressure overload. *Hypertension*.

[23] Zeng C., Zhong P., Zhao Y. (2015). Curcumin protects hearts from FFA-induced injury by activating Nrf2 and inactivating NF-*κ*B both in vitro and in vivo. *Journal of Molecular and Cellular Cardiology*.

[24] Hollman P. C. H., Katan M. B. (1999). Health effects and bioavailability of dietary flavonols. *Free Radical Research*.

[25] Gutiérrez-Venegas G., Luna O., Arreguín-Cano J., Hernández-Bermúdez C. (2014). Myricetin blocks lipoteichoic acid-induced COX-2 expression in human gingival fibroblasts. *Cellular & Molecular Biology Letters*.

[26] Lee Y. S., Choi E. M. (2010). Myricetin inhibits IL-1*β*-induced inflammatory mediators in SW982 human synovial sarcoma cells. *International Immunopharmacology*.

[27] Chen L., Huang J., Ji Y. X. (2017). Tripartite motif 8 contributes to pathological cardiac hypertrophy through enhancing transforming growth factor *β*-activated kinase 1-dependent signaling pathways. *Hypertension*.

[28] Li C. Y., Zhou Q., Yang L. C. (2016). Dual-specificity phosphatase 14 protects the heart from aortic banding-induced cardiac hypertrophy and dysfunction through inactivation of TAK1-P38MAPK/-JNK1/2 signaling pathway. *Basic Research in Cardiology*.

[29] Ji Y. X., Zhang P., Zhang X. J. (2016). The ubiquitin E3 ligase TRAF6 exacerbates pathological cardiac hypertrophy via TAK1-dependent signalling. *Nature Communications*.

[30] Lamothe B., Besse A., Campos A. D., Webster W. K., Wu H., Darnay B. G. (2007). Site-specific Lys-63-linked tumor necrosis factor receptor-associated factor 6 auto-ubiquitination is a critical determinant of I kappa B kinase activation. *The Journal of Biological Chemistry*.

[31] Yin Q., Lin S. C., Lamothe B. (2009). E2 interaction and dimerization in the crystal structure of TRAF6. *Nature Structural & Molecular Biology*.

